# News and views (11&12): Topological Modes Unshackled; 2022 JPS Nishina Award; 50th, 51st & 52nd AAPPS Council Meetings, 11th AAPPS Ordinary General Council Meeting

**DOI:** 10.1007/s43673-022-00066-z

**Published:** 2022-12-22

**Authors:** AAPPS Bulletin

**Affiliations:** Association of Asia Pacific Physical Societies, Pohang, South Korea

## Topological Modes Unshackled by Guancong Ma

Topological matters have been a focus of research across many realms of physics, ranging from quantum electronics [1, 2] and photonics [3], to classical systems such as acoustics and mechanics [4]. Such a long-lasting interest owes not only to the physical and mathematical elegance in the theories for topological matters but also to the immense practical possibilities of topological modes. These topological modes are robust against disorders because their existence is protected by the topology of the bulk energy bands—global properties of the Bloch wavefunctions that are insensitive to many local perturbations in the lattice. In addition, topological modes can become unidirectional transfer channels that are immune to backscattering, making them ideal for energy or information transfer applications.

However, we all know well that there is no free lunch in the world. A price has to be paid in order to access the fantastic properties of topological modes. All topological modes are localized modes in character, i.e., their wavefunctions are maximum at boundaries or defects of a lattice and the wavefunctions exponentially decay toward the bulk. Thus, to access a topological mode, one has to build a lattice that spans at least one additional spatial dimension. Some even regard this to be the “Achilles’ heel” of topological matters, for any device relying on topological modes must be bulky in size and costly to produce.

In a recent work, physicists at Hong Kong Baptist University leveraged non-Hermitian skin effects to turn the wavefunctions of topological modes into a fully extended mode across the entire lattice, effectively “unshackling” them from the boundary [5]. This breakthrough broadens and deepens the current understanding of the topological modes and their application potentials.

Non-Hermitian skin effects, in a recent discovery, tie non-Hermitian degrees of freedom with band topology. Traditionally, quantum mechanical systems are described by Hermitian operators because they produce a real spectrum and an orthonormal set of eigenvectors, which are desirable mathematical properties for describing close systems, in which energy and probabilities are conserved. In contrast, non-Hermitian operators generically have complex-value eigenvalues, making them useful in describing some scenarios in which a system is “open,” i.e., a system that exchanges energy with its surroundings [6, 7]. Physicists have found that when considering the non-Hermitian skin effect in the contexts of topological matters, the bulk-boundary correspondence, which allows the prediction of topological transitions by considering only a single unit cell under the periodic boundary condition, no longer gives sensible results [8–10]. Instead, in the presence of open boundaries, the wavefunctions of the majority of the bulk modes appear to “pile up” at an open boundary, becoming “skin modes.” In other words, it turns out Bloch wavefunctions can no longer predict the open-boundary wavefunctions of the bulk modes. Hence, there is no reason bulk-boundary correspondence should hold.

**Fig. 1:**
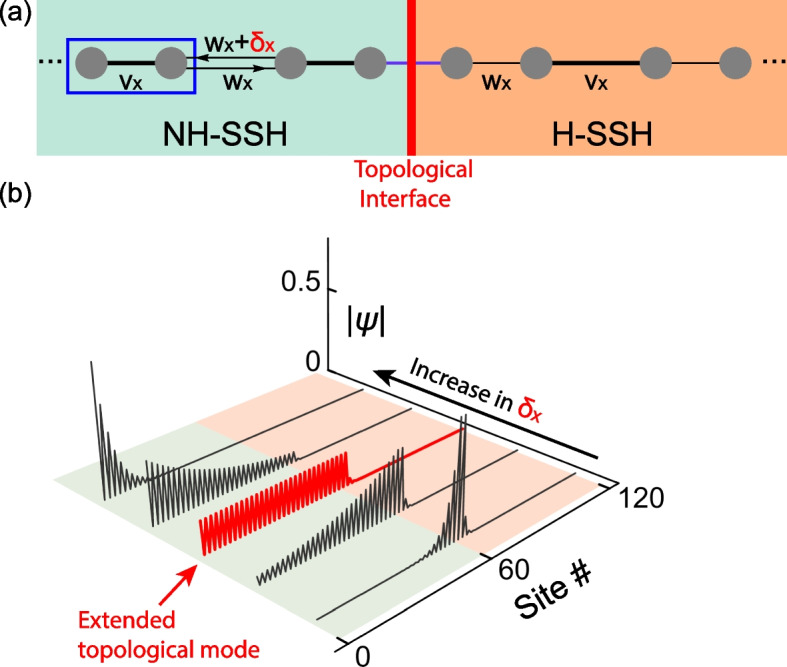
**a** A one-dimensional topological interface (red line). The left part (green) is topologically trivial but is non-Hermitian, where the non-reciprocal hopping term *δ*_*x*_ is responsible for the non-Hermiticity. The right part (orange) is topological and Hermitian. Here, the hopping terms are |*v*_*x*_| > |*w*_*x*_|. **b** The magnitude of the wavefunction of the topological mode is denoted as |*ψ*|. The localization property of the topological mode is drastically affected by *δ*_*x*_. It can be fully extended in the non-Hermitian half chain (red)

Wang et al. demonstrated in their work that similar physics can conversely be used to modify the wavefunctions of topological modes [5]. Non-Hermiticity is introduced as non-reciprocal hopping between unit cells, i.e., *δ*_*x*_ in Fig. 1a. And when the conditions are right, it can entirely counter the exponential decay of the topological modes in the bulk lattice, rendering the topological modes extended (Fig. 1b). Wang et al. also showed that the topological modes can even morph into almost arbitrary shapes by engineering the distribution of the non-Hermiticity. These results challenge the common belief that the topological modes are always localized at the boundaries of the system or the defects. The said effects are successfully realized in non-Hermitian mechanical lattices.

The underlying physics of reshaping the topological modes is general because both the non-Hermitian skin effect and topological matters are realizable in many realms. We envision that these results may empower a wide range of topological applications, e.g., large-area single-mode topological lasers and coherent topological beam splitters.

## Citation for the 2022 (the 10th) Nishina Asia Award by JPS


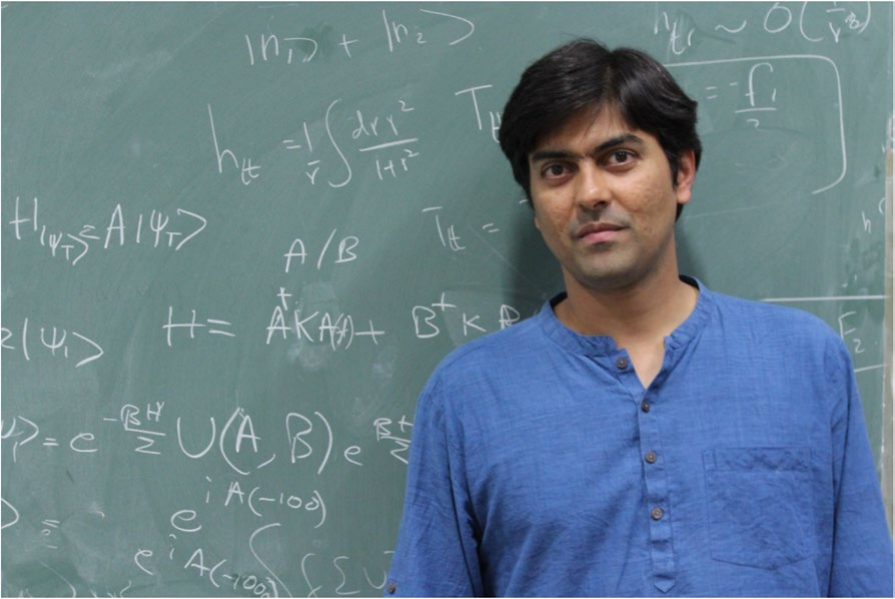


Dr. Suvrat Raju

Professor, International Centre for Theoretical Sciences, Tata Institute of Fundamental Research, Bangalore, India

*For his original and influential insights into the resolution of the black hole information paradox and the principle of holography in quantum gravity.*

The black hole information paradox has been a longstanding problem in quantum gravity since the discovery of Hawking radiation and black hole evaporation in the 1970s. Although some of the important questions regarding quantum black holes have been clarified from the perspective of string theory, the information paradox, which jeopardizes quantum unitarity, remains a critical issue. Dr. Raju made an original and essential contribution to the subject by pointing out a characteristic feature of the quantum Hilbert space of a black hole with Hawking radiation, which can describe the states inside and outside of the black hole horizon at the same time. His proposal has significantly influenced recent research on the black hole information paradox, such as the island proposal, and has drawn attention to the importance of the non-split property of the black hole Hilbert space.

### Detailed Description

Understanding quantum gravity is one of the most fundamental and difficult problems in high-energy physics. While string theory has provided an elegant solution to the well-known difficulty of uncontrollable ultraviolet divergence encountered in quantizing gravity, quantum gravity still poses many deep questions that have remained unanswered.

The complexity of quantum gravity perhaps started with the recognition that, surprisingly, general relativity exhibits the structure of thermodynamics. As argued by J. Bekenstein, a black hole is a thermodynamic object with (in general) finite temperature and carries an entropy proportional to the area of its horizon (not to the volume of a black hole as one would expect). Moreover, S. Hawking found that a black hole, when the quantum effect is understood in a semi-classical approximation, can emit quanta, the spectrum of which is exactly that of radiation emitted by a black body.

These findings implied that quantum black holes must have truly enigmatic and troublesome properties: (i) There appears to be a violation of unitarity in the time evolution from an initial pure state that collapses to a black hole, which ends up in a mixed state that emits thermal radiation. (ii) This would imply that the information thrown into a black hole will be eternally lost, in acute contradiction with the principles of quantum mechanics. (iii) As the entropy measures the logarithm of the number of possible microscopic quantum states under a specified macroscopic condition, how can it be proportional to the area when the black hole is a three-dimensional object?

The last question prompted G. ’t Hooft and L. Susskind, among others, to put forward a bold idea that all the information is stored on the horizon just as in holography. Further, J. Maldacena made this idea much more precise with concrete examples and proposed the “bulk/boundary duality”, where a gravitational theory in the bulk of spacetime can be equivalent to a suitable non-gravitational theory defined on the boundary. If this conjecture is fully substantiated, the serious problems listed above may be solved, provided that the boundary theory respects unitarity. Namely, the corresponding bulk theory with the emergent gravitational degrees of freedom will also be unitary and, if treated exactly, the apparent problem of information loss by a black hole should disappear.

However, upon close examination, it appears that a naive application of this promising idea gives rise to another serious problem. S. Mathur and J. Polchinski and his collaborators independently pointed out that if one assumes the unitarity of the boundary theory, a region with highly excited quanta, dubbed a “firewall”, would form in the neighborhood of the black hole horizon, and the celebrated equivalence principle of general relativity would break down. Spacetime would then end at the horizon and the interior of a black hole together with the information stored within would not be described by the boundary theory.

It is in this context that Dr. Raju, together with K. Papadodimas, presented a novel idea that would resolve this impasse. They made use of the thermo-field double description of field theory at finite temperature, which uses a highly entangled system consisting of two copies of the original system. In the limit of large N for the typical boundary theory with SU (N ) gauge symmetry, they demonstrated that one can construct an effective operator, in terms of a non-trivial combination of operators of the first copy describing the system outside the horizon, which, when acted on the thermo-field double state, can create a state in the second system in a characteristic state-dependent way. They further argued that this second copy can be interpreted as the Hilbert space describing the interior of a black hole. Thus, their idea materialized the notion of so-called “black hole complementarity” and asserts that the information inside a black hole can already be fully encoded in the system outside.

Although their work aroused a substantial amount of interest, its essential importance started to become apparent only rather recently. In the past couple of years, substantial progress has been made by the recognition that the failure of unitarity inferred from the original computation of Hawking is due to the omission of an important contribution from a semi-classical saddle, called an “island,” roughly inside the black hole horizon. Although this has been established in the path-integral formulation, it appears quite significant that the degrees of freedom of the island is made up of complicated state-dependent combinations of Hawking radiation modes. This, of course, is highly reminiscent of the Papadodimas-Raju construction.

The deep influence of the work by Dr. Raju, including those after the Papadonimas-Raju paper, can also be seen in the recent, significant work by S. Leutheusser and Liu, which tries to create the emergent “time” in the interior of a black hole. It was duly emphasized that of intrinsic importance is the so-called non-split property of the Hilbert space, namely that the Hilbert space of a black hole cannot be factored as the tensor product H_inside_ ⊗ H_outside_.

Their work in turn prompted Witten and his collaborators to study a change in the properties of the von Neumann algebra of observables when the 1/N gravitational corrections from the large N limit are taken into account in the holographic framework. It should be recognized that these promising developments can be said to have their roots in the work of Dr. Raju and K. Papadodimas.

We must mention also that more recently, Dr. Raju has produced further influential contributions to the holographic principle in quantum gravity and the black hole paradox by analyzing these problems for more realistic situations. One such work is the study of holography in four-dimensional flat spacetime, performed with his collaborators. They showed that, at least for massless excitations, all the information present at future null infinity is also present near its past boundary. This original proposal for flat space holography is a significant step toward understanding quantum gravity in a realistic spacetime.

Another recent work of Dr. Raju and his collaborators is a critical assessment of the “island proposal” for the recovery of unitarity mentioned above. They pointed out that such a proposal is applicable only in the presence of a non-gravitational bath and massive gravitons. In addition, as a more robust approach to understanding the unitarity of black hole evaporation, it was emphasized that the aforementioned non-split property of the Hilbert space and the associated structure of the von Neumann algebra of the observables must be duly taken into account in the presence of gravity.

## Report on the 50th to 52nd Council Meetings by AAPPS

### 50th AAPPS Council Meeting

The 50th Council Meeting of the Association of Asia Pacific Physical Societies (AAPPS) was held in a hybrid format from 10:00 a.m. to 11:34 a.m. (UTC+9hr) on August 21, 2022. The in-person venue was the faculty meeting room in the physics department at Yonsei University, Seoul, which was connected online via a Zoom session hosted by the Asia Pacific Center for Theoretical Physics (APCTP). The participants were Jun'ichi Yokoyama (president, in person), Hyoung Joon Choi (vice president, in person), Nobuko Naka (secretary), Keun-Young Kim (treasurer, in person), Gui-Lu Long (ex officio member as a former president), and council members Jodie Bradby (Australian Institute of Physics (AIP)), Xiu-dong Sun (the Chinese Physical Society, Beijing), Tao Xiang (the Chinese Physical Society, Beijing), Ruiqin Zhang (the Physical Society of Hong Kong, in person), Mio Murao (the Physical Society of Japan (JPS)), Akira Yamada (the Japan Society of Applied Physics (JSAP)), Woo-Sung Jung (the Korean Physical Society (KPS), in person), Kurunathan Ratnavelu (Malaysian Institute of Physics), Rajdeep Singh Rawat (Institute of Physics Singapore), Meng-Fan Luo (the Physical Society located in Taipei), and Nguyen Quang Liem (Vietnam Physical Society). Present as observers were Kadyr Gulamov (president of the Council of Uzbekistan Physicists, in person), Xun-Li Wang (president of the Physical Society of Hong Kong), Mirim Lee (academic support staff, APCTP, in person), Sojin Park (academic support staff, APCTP, in person), and Kyeongtak Ryu (academic support staff, APCTP, in person). Ying-Jer Kao (president of the Physical Society located in Taipei, in person) was present as the proxy on behalf of council member Fu-Jen Kao (the Physical Society located in Taipei).

(1) Secretary Naka reported the presence of 17 council members (including the proxy) out of 17 council members, and the quorum was declared as fulfilled. She announced that the minutes of the 49th Council Meeting were circulated by e-mail.

(2) President Yokoyama opened the 50th Council Meeting and welcomed the participants. The agenda was adopted as prepared by the president.

(3) Vice President Choi reported on the status of preparations for APPC15. He announced that the welcome reception will be held on the metaverse platform from 6 p.m. on August 21, 2022. On the same platform, AAPPS and *AAPPS Bulletin* (*AB*) will have respective booths for exhibition. Choi presented the list of committee chairs and members. The sessions are categorized into 14 subjects covering all areas of physics. Choi explained that the Women-in-Physics sessions are open to everyone, including those who did not pay the registration fee. At the next APPC, the Women-in-Physics sessions may be shifted from ordinary to special topics. Finally, we have 500 invited talks, 300 contributed talks, and approximately 200 posters; there are slightly more than 1000 total contributions. Choi stated that these numbers tell us that we have achieved our original goal of making APPC15 successful.

The schedule for two plenary talks from abroad was modified to Tuesday and Wednesday. We will have poster sessions on Monday through Wednesday and not on Thursday and Friday. The special sessions include the Global Physics Summit (hybrid), the Role of Physics in the Green Economy (hybrid), the AAPPS-APCTP CN Yang Award ceremony (hybrid), and the awardee’s talk session. In the editors’ session on Wednesday, editors of the *Journal of the Korean Physical Society*, *AB*, and *Nature Physics* will each deliver 20 min-long presentations including questions and answers. These special sessions will be open to the public.

Rajdeep Singh Rawat raised the question of how an individual speaker could access the sessions. Choi explained that participants are supposed to login to the APPC15 website, in order to access the Zoom links. The steps are detailed in the guidelines posted on the website’s “News & Notice” page. Rawat suggested announcing the information. Choi also announced that respective chairpersons are responsible for timekeeping.

(4) Rawat, the chair of the selection committee of the CN Yang Award 2022, made a briefing on the selection process this year. He explained the eligibility for nomination and criteria (novelty and originality, independence, and impact) for evaluation. This year’s three winners are all young researchers with prominent achievements and they are expected to become leaders in the Asia Pacific region. The nomination channels are not limited to AAPPS member societies and APCTP member entities but individual nominations are also possible if they come from attendees of the immediate past APPC.

Altogether, there were 31 nominees affiliated with different sessions of APPC15. Twenty-seven nominations were from member societies and AAPPS divisions, while the remaining four nominations were from individuals. There was a widespread distribution over countries and regions. Among them, 19 nominations were sent to four AAPPS divisions for preselection. For the other 12 nominations from nine different fields not belonging to AAPPS divisions, each candidate was reviewed by from 2 to 4 sub-selection committee members from AAPPS and APCTP. In total, 18 candidates were recommended for the final selection process.

The final selection was a two-step process, basically examining the results of prescreening, and was made by the selection committee, which was comprised of the committee chair and 12 other members (four division chairs, four members nominated by the AAPPS president, and another four nominated by the APCTP president). One more evaluation round was made for the final selection, based on the ranking of the scores of the candidates and by referring to the comments from the reviewers in the sub-selection committee. After receiving the scores from the selection committee members, the ranks of average scores and statistics of the rank-based scores were carefully examined. Finally, at a Zoom meeting, a unanimous agreement was reached by the selection committee to select the top three candidates as winners. The selection committee discussed and confirmed that all these winners represent different member societies and have no overlap of subjects with those of recent past winners. The winners are from the fields of applied physics, quantum information, astrophysics, cosmology, and gravitation, respectively. The decision was also approved by the AAPPS president and the APCTP president.

Rawat appreciated all those who made nominations of high-profile young researchers (as that made this award extremely selective), members of sub-selection and selection committees for evaluation and decisions, the AAPPS and APCTP presidents for support and guidance, Prof. Fu-Jen Kao for sharing experience and expertise as the past chair of the selection committee, as well as Ms. Dayoung Yang and APCTP support staff for their excellent works including enormous email communications.

Xun-Li Wang expressed his great pleasure of having a recipient of such a prestigious award this year from the Physical Society of Hong Kong. He added that this would be the first time that a member of the Physical Society of Hong Kong receives the CN Yang Award. Yokoyama wondered if there were any female candidates. Rawat responded that it is a good point to examine. He commented that the selection committee had a fair fraction of female members.

(5) Naka explained that the election of new council members will be conducted at the 11th ordinary general meeting (OGM). She announced that all official representatives from respective member societies, both on-site and off-site, will use electronic voting ballots. Fifteen new council members will be elected among 18 candidates nominated from 13 member societies. With the help of Treasurer Kim, she sent a notice regarding Clause 7.3 of the Constitution that member societies with unpaid fees have no voting rights at the present OGM. It was confirmed that official representatives from 14 member societies have the right to vote at the 11th OGM. Yokoyama reminded the official representatives of the election procedure by citing Bylaws 9-12. He commented that it is a very good thing for AAPPS that this time we have a larger number of candidates than the number of vacancies.

Yokoyama also reported on the preparation status of the 11th OGM, whose agenda will include the election, discussions, and the decision regarding amending the Constitution, the financial report, and a report regarding the status of *AB*.

(6) Treasurer Kim gave a brief report on the financial status of AAPPS.

The carryover at the beginning of the year 2021 was 63,394,502 KRW. There was only a single expense of the establishment fee for the Division of Condensed Matter Physics in 2021. The carryover to the year 2022 was 74,052,140 KRW. The expenses that were incurred were the support of $1500 USD to the Siam Physics Congress held in Thailand, $2000 USD to the Nepal Physical Society, and $1500 USD to the Council of Uzbekistan Physicists. Nepal and Uzbekistan have used the money for the membership fees for 2009–2018 and 2022–2024, for their respective societies. The total balance is $67,014 USD in addition to the Leo Koguan Foundation’s $36,500 USD.

Kim showed the overall membership fee status. Yokoyama expressed that he is glad that India and the Philippines have recently come back to AAPPS. He reported that he received a message from Kazakhstan and that the fee would be paid around September 2022. Nguyen Quang Liem commented that the Vietnam Physical Society already transferred the fee for 2020. Kim also explained the contributions for *AB* and others from five societies, and extra accumulated support from APCTP of 491,470 USD. The Division of Astrophysics, Cosmology and Gravitation (DACG) has supported the domain fee of $113 USD until 2025.

Yokoyama summarized that we have not spent much money because we conducted research activities and communication through virtual platforms during the pandemic.

(7) Gui-Lu Long, the editor-in-chief of the *AAPPS Bulletin* (*AB*) reported on the current status of *AB*.

*AB* is the flagship journal of AAPPS. The first issue was published in 1991, already 32 years ago. In 2010, the journal stopped publication because of financial and management difficulties. A revival occurred with the help of APCTP and KPS, and a memorandum among three entities (AAPPS, APCTP, and KPS) to cooperatively publish *AB* was signed. Publication restarted in August 2011 and since then 67 issues have been published. Normally, the editors have face-to-face meetings once a year and video meetings every month. Because of Covid-19, however, on-site meetings were suspended.

The budget’s balance is $149,604 USD. Each issue costs $2000 USD for printing and approximately 1700 copies are sent out each time to related institutions, individuals, and cooperate members. The cost is covered by *AB* contributions of $5000 USD each provided by the Chinese Physical Society, Beijing, KPS, the Physical Society located in Taipei, and JPS. The 10 cooperate members pay $1500 USD each for advertisement and receive 50 copies per issue, and the other three cooperate members pay $750 USD each and receive 25 copies per issue. The total income is $17,250 USD. The Article Publication Charge (APC) is $1870 USD for each of all published articles.

Long explained that they began to contact major publishers in 2020. A contract was made to publish all items online on the Springer-Nature website from 2021 and in print while maintaining the existing website, which was renewed last month. The articles are categorized into Research and Reviews, Research Highlights, and News and Views, the last one of which replaces the previous Society News.

Long emphasized that the citations are significantly improving. The average citations of articles published in 2018 and 2019 were around 1 while it was nearly 7 for articles published in 2021. The citations are expected to reach a value close to 10 at the end of 2022, verifying that *AB* proceeds in the right direction. Long acknowledged APCTP for its great efforts and contributions. Long added that the editorial board has been expanded and now consists of one editor-in-chief, three deputy editor-in-chiefs, 15 senior editors, and 49 editors. Each year, starting from June, 1/3 of the editors are replaced routinely.

Long stated that *AB* will continue publishing high-quality reviews and research papers. Three plans were highlighted for the future: (i) publication of 40–100 articles each year, targeted from now to 2025; (ii) promotion of the journal through the range of Springer-Nature channels; and (iii) indexing *AB* in two major indexes, i.e., SCOPUS and the Web of Science. Long explained that if *AB* could publish 100 or more free submissions per year, the authors will pay $200,000 USD in a year, and *AB* can run self-sustainably.

Yokoyama asked whether the eight original articles published in 2022 were all invited papers and indeed the APC was entirely covered by APCTP. Long responded yes and explained that *AB* needs to negotiate with APCTP when the number of articles is increased to over 40 per year. Kurunathan Ratnavelu congratulated *AB* for its success. He wondered whether the cooperate members must be universities, research institutes, multinational companies, or foundations. Long explained that Prof. Nagamiya and Prof. Namkung invited many cooperate members, for which *AB* make advertisement for them. Recently, JPS paid $3600 USD and the advertisement article was published in Research Highlights. Ratnavelu stated that he will try to discuss if the Malaysian Institute of Physics could make a contribution, and Long and Yokoyama appreciated the offer. Rawat thanked Long for his hard work and congratulated the achievements of *AB*. He suggested Long could invite participants of APPC15 to provide submissions.

Yokoyama wondered whether the proceedings of APPC15 will be published. Long commented that in order to be published in *AB*, there should be a review process. Choi explained that to publish the proceedings in a journal, we would have to reject more than half of the papers. Instead, the proceedings will be published in an online book with DOI numbers from Springer Nature, which will not be indexed in a database.

(8) Yokoyama reported that he has not received any request for changes on the draft of the Code of Conduct after it was distributed at the previous council meeting on July 28, 2022. Jodie Bradby expressed her support by stating that the draft looks good. The proposed draft was adopted.

(9) Yokoyama expressed his sincere gratitude to Liem and Fu-Jen Kao, who have served as council members for three terms. Liem stated that Kao and he have tried their best to contribute to AAPPS. He hopes the next representative from the Vietnam Physical Society will be elected and will continue to participate actively in AAPPS. He discussed with President Nguyen Dai Hung that the Vietnam Physical Society will work more to host conferences in the future, and he looks forward to seeing other council members in Vietnam. Yokoyama stated that he is also grateful to Long, who served as vice president and president and is presently serving as editor-in-chief. Long will be leaving the council in December, although he will stay as the editor-in-chief of *AB*.

Yokoyama closed the meeting.

### 51st AAPPS Council Meeting

The 51st Council Meeting of the Association of Asia Pacific Physical Societies (AAPPS) was held in a hybrid format from 3:40 p.m. to 4:14 p.m. (UTC+9hr) on August 21, 2022. The in-person venue was the faculty meeting room in the physics department at Yonsei University, Seoul, which was connected online via a Zoom session hosted by the Asia Pacific Center for Theoretical Physics (APCTP). The participants were Jun’ichi Yokoyama (current president, in person), and new council members Nicole F. Bell (Australian Institute of Physics (AIP)), Xiu-dong Sun (the Chinese Physical Society, Beijing), Tao Xiang (the Chinese Physical Society, Beijing), Ruiqin Zhang (the Physical Society of Hong Kong, in person), Mio Murao (the Physical Society of Japan (JPS)), Shen Qing (the Japan Society of Applied Physics (JSAP)), Hyoung Joon Choi (the Korean Physical Society (KPS), in person), Jae-Hyung Jeon (KPS, executive director of APCTP, in person), Keun-Young Kim (KPS, in person), Kurunathan Ratnavelu (Malaysian Institute of Physics), Rajdeep Singh Rawat (Institute of Physics Singapore), Meng-Fan Luo (the Physical Society located in Taipei), Vu Dinh Lam (Vietnam Physical Society), and Kadyr Gulamov (Council of Uzbekistan Physicists, in person). Present as observers were Nobuko Naka (current secretary), Mirim Lee (academic support staff, APCTP, in person), Sojin Park (academic support staff, APCTP, in person), and Kyeongtak Ryu (academic support staff, APCTP, in person). New council member Narayan Prasad Chapagain (the Nepal Physical Society) was absent.

(1) Secretary Naka reported the presence of 15 new council members out of 16 members. The quorum was declared as fulfilled.

(2) Yokoyama opened the 51st Council Meeting and welcomed the participants, including five newly elected members. He explained that the term of outgoing council members is almost finished. Although the new term officially starts in January 2023, he wishes to invite all new council members to join whenever council meetings will be held. The new council members and other participants introduced themselves. The agenda was adopted as prepared by the president.

(3) Yokoyama explained the procedure of the elections of the next president and vice president. According to Bylaws 15–19, a simple majority of the vote cast is required to be declared elected, and a candidate who received the fewest number of votes or voluntarily withdraws will be eliminated in the second round.

Yokoyama called for nominations of candidates for the position of the next president. Rajdeep Singh Rawat nominated Hyoung Joon Choi, which was seconded by Kurunathan Ratnavelu, Tao Xiang, and Kadyr Gulamov. There were no other nominations, and an open vote of confidence was conducted. Choi was elected as the next president by common consent. Choi made an address that he feels a heavy burden in having this position, particularly after working hard in this and last years for APPC15 as the program chair. To fulfill his responsibilities during the next term of 3 years, he strives to make the relationship among member societies in AAPPS even stronger and to encourage more AAPPS activities.

Subsequently, Yokoyama called for nominations of candidates for the position of the next vice president. Ruiqin Zhang and Kurunathan Ratnavelu nominated Tao Xiang, which was seconded by Xiu-dong Sun and Vu Dinh Lam. There were no other nominations, and an open vote of confidence was conducted. Xiang was elected as the next vice president by common consent. Xiang expressed his thanks to council members for trusting him. He considers his duty is to promote more cooperation among council members in AAPPS and, in the near future, to help AAPPS become one of the most influential societies in the world, like its counterparts, i.e., the American Physical Society and the European Physical Society.

(4) President-elect Choi announced that the next secretary and treasurer will be appointed later in due course. His decision will be circulated by e-mail for approval of council members.

(5) Kadyr Gulamov made a speech on the occasion of the real presence of the chairman of the Council of Uzbekistan Physicists at the meeting venue. He emphasized the importance of the visibility of the association and the effort to attract money for the involvement of young researchers and scientists. Choi responded that he will seriously consider the possibility of having support from companies. Yokoyama commented that the International Union of Pure and Applied Physics (IUPAP) does start searching for industrial members.

Yokoyama closed the meeting.

### 52nd AAPPS Extended Council Meeting

The 52nd Extended Council Meeting of the Association of Asia Pacific Physical Societies (AAPPS) was held in a hybrid format from 3:40 p.m. to 6:03 p.m. (UTC+9hr) on August 21, 2022. The in-person venue was the faculty meeting room in the physics department at Yonsei University, Seoul, which was connected online via a Zoom session hosted by the Asia Pacific Center for Theoretical Physics (APCTP).

The participants were Jun’ichi Yokoyama (current president, in person), Hyoung Joon Choi (current vice president, in person), Nobuko Naka (current secretary), Keun-Young Kim (current treasurer, in person), Gui-Lu Long (ex officio member as a former president and editor-in-chief), and current council members Xiu-dong Sun (the Chinese Physical Society, Beijing), Tao Xiang (the Chinese Physical Society, Beijing), Ruiqin Zhang (the Physical Society of Hong Kong, in person), Mio Murao (the Physical Society of Japan (JPS)), Akira Yamada (the Japan Society of Applied Physics (JSAP)), Woo-Sung Jung (the Korean Physical Society (KPS), in person), Kurunathan Ratnavelu (Malaysian Institute of Physics), Rajdeep Singh Rawat (Institute of Physics Singapore), Meng-Fan Luo (the Physical Society located in Taipei), and Nguyen Quang Liem (Vietnam Physical Society). Current council members Jodie Bradby (Australian Institute of Physics (AIP)) and Fu-Jen Kao (the Physical Society located in Taipei) were absent. Ying-Jer Kao (president of the Physical Society located in Taipei, in person) was present as a proxy on behalf of Fu-Jen Kao. Attended as new (and not continuing) council members were Shen Qing (JSAP), Jae-Hyung Jeon (KPS, executive director of APCTP, in person), Vu Dinh Lam (Vietnam Physical Society), and Kadyr Gulamov (Council of Uzbekistan Physicists, in person). New council members Nicole F. Bell (AIP) and Narayan Prasad Chapagain (the Nepal Physical Society) were absent.

Attended as official representatives of member societies were Gui-Lu Long* (proxy of Jie Zhang, the Chinese Physical Society, Beijing), Setsuko Tajima (president of JPS), Akira Yamada* (proxy of Toshiro Hiramoto, JSAP), Tae Won Noh (president of KPS, in person), Tou Teck Yong (president of the Malaysian Institute of Physics), Michael Francis Ian Vega II (proxy of Elmer Estacio, Physics Society of the Philippines), Rajdeep Singh Rawat* (proxy of Christian Kurtsiefer, Institute of Physics Singapore), Ying-Jer Kao* (president of the Physical Society located in Taipei, in person), Bobomurat Ahmedov (proxy of Kadyr Gulamov, Council of Uzbekistan Physicists), and Nguyen Dai Hung (president of the Vietnam Physical Society) [note: asterisks indicate those who attended also as a council member or a proxy.] Official representatives Kirrily Rule (proxy of Sven Rogge, AIP), Xun-Li Wang (president of the Physical Society of Hong Kong), Vandana Nanal (proxy of S. Ramakrishnan, Indian Physics Association), T.A. Kozhamkulov (president of the Kazakh Physical Society), Nilam Shrestha Pradhan (president of the Nepal Physical Society), Riaz Ahmed (president of the Pakistan Physical Society), and Boonrucksar Soonthornthum (president of the Thai Physics Society) were absent.

Present as observers were Je-Geun Park (chair of the Division of Condensed Matter Physics (DCMP)), Weiping Liu (chair of the Division of Nuclear Physics (DNP)), Sang Pyo Kim (chair of the Division of Astrophysics, Cosmology and Gravitation (DACG)), Baonian Wan (chair of the Division of Plasma Physics (DPP)), Yunkyu Bang (president of APCTP, in person), Mirim Lee (academic support staff, APCTP, in person), Sojin Park (academic support staff, APCTP, in person), and Kyeongtak Ryu (academic support staff, APCTP, in person).

(1) Secretary Naka reported the presence of 16 current council members (including a proxy) out of 17 members, and 14 new council members out of 16 members. The quorum was declared as fulfilled.

(2) President Yokoyama opened the 52nd Extended Council Meeting and welcomed the participants. The agenda was adopted as prepared by the president.

(3) Yokoyama reported that the new council members, who will serve from 2023 to 2025, were elected at the 11th Ordinary General Meeting (OGM). The list of 15 new council members was confirmed. He also explained that Hyoung Joon Choi and Tao Xiang were elected for the next president and vice president, respectively, at the 51st Council Meeting. The current president will join the new council as an ex officio.

(4) All participants introduced themselves.

(5) Vice President Choi reported on the status of APPC15. The opening session is planned to start from 9:00 a.m. on Monday, August 22, 2022. The lists of chairs and the committee members were shown. The number of International Program Committee members is approximately 200, where typically 10 members represent each of the 14 subjects. The topics include all areas of physics and Women-in-Physics. He commented that the Women-in-Physics sessions could be special sessions at the next APPC, instead of ordinary sessions. 15 plenary talks, about 500 invited talks, more than 300 contributed talks, and slightly less than 200 posters will be delivered at APPC15. Choi commented that the number of poster presentations was quite small this time.

The special sessions include the Global Physics Summit, the Role of Physics in the Green Economy, the AAPPS-APCTP CN Yang Award ceremony, and the awardee’s talk session on Monday. In the Global Physics Summit, the president of the International Union of Pure and Applied Physics (IUPAP), the president of the European Physical Society (EPS), and the president of the American Physical Society (APS) will deliver presentations. The president of IUPAP will also deliver a presentation in the Role of Physics in the Green Economy session and stay in this session for 3 h. On Tuesday, the Women-in-Physics session starts in the morning. The editor session will be held on Wednesday, in which editors of the *Journal of the Korean Physical Society*, *AAPPS Bulletin* (*AB*), and *Nature Physics* will each deliver 20 min-long presentations including questions and answers.

Sang Pyo Kim conveyed a question about the total number of registrations. Choi responded that it was more than 1000. Weiping Liu suggested making the registration free for those who cannot attend in person, or the conference sends out some souvenirs, such as printed conference proceedings and so on. Choi responded that they could not make such plans. The proceedings will be published online by Springer Nature and accessed only by the registered participants. Choi stated that he will inform Liu after investigating the contract with Springer Nature. He added that the special sessions on Monday through Wednesday are open to everyone regardless of the registration, and he hopes everyone enjoys the conference online.

(6) Yokoyama explained that he made a call to member societies for a proposal to host the next APPC. The Chinese Physical Society, Beijing, submitted a proposal to hold APPC16 in 2025, which was endorsed by the council at the 48th Council Meeting. Tao Xiang stated that he will be the main organizer and he and Xiu-dong Sun will do their best to make APPC16 as successful as older APPCs. He welcomes participants in Beijing on site without hybridization and in association with the OGM. In previous council meetings, it was discussed if the interval of APPCs can be shorter or if the conference can be combined with the local society’s events. To further discuss such a matter in a long-time scale, the establishment of a new working group in the new council was proposed. Xiang explained that because the Chinese Physical Society, Beijing, organizes annual conferences in September, he would be able to negotiate with the society whether special AAPPS sessions could be incorporated into the annual conference of the society.

Yokoyama wondered if such special sessions at a meeting of the Chinese Physical Society, Beijing, could be conducted in English, and if so, whether they could make some local events more international. Ying-Jer Kao explained that all the talks are given in English at the meetings of the Physical Society located in Taipei. Yokoyama commented that the past APPC held in Australia was combined with their congress. Apart from APPCs held every 3 years, all four divisions organize annual meetings of their own, which may be combined with member societies’ annual meetings.

Setsuko Tajima explained that JPS has not been able to hold in-person meetings for 3 years. The first in-person meeting, after the 3-year suspension due to the pandemic, will take place this fall. She stated that through this truly difficult period we have learned about some of the advantages of holding online meetings. Among the biannual meetings, JPS has decided to hold all the Spring Meetings online and is planning to hold only the Fall Meetings in person. The online format is more convenient to organize international sessions as is it easier to invite more foreign researchers. She clarified that not full sessions of meetings, but focused sessions could be international. Even during the pandemic, the nuclear physics division recently organized a joint symposium with KPS. As seen in this example, the organization of international sessions is possible if researchers from specific fields agree with each other to do so. She considers that holding an AAPPS joint symposium would also be possible through the initiative of some divisions inside JPS.

Liu explained that the Chinese Physical Society, Beijing, had a joint meeting with the nuclear physics division of JPS. Normally before the pandemic, they had in-person meetings in domestic China. They started to involve Korean and Japanese colleagues by implementing a hybrid format, and similar hybridization hosted by Korea or by Japan instead of China became also possible, which increased the number of participants from other areas of Asia. Liu hopes that the situation gets much better so that in-person meetings become once again, the standard.

Tae Won Noh stated that the KPS usually organizes two annual meetings attended by 1500–2000 participants. At each meeting, a few plenary talks, pioneer sessions, focused sessions, poster sessions, etc., are organized. Plenary talks, pioneer sessions, and some focused sessions are given in English. This year marks the 70th anniversary of the KPS. Noh stated that it would be easy to involve AAPPS sessions in the KPS meetings.

Tou Teck Yong explained that the Malaysian Institute of Physics has been hosting joint meetings with AAPPS quite regularly. According to the conference topics, they have invited people from some specific societies, such as computational physics or synchrotron radiation and intense laser facility, to international or domestic physics conferences. At the physics conference this year, semiconductor researchers from the industry were invited as keynote speakers, which fostered more innovation and excitement for students.

Michael Francis Ian Vega II stated that the Physics Society of the Philippines has been holding meetings online. The annual meetings were originally held in July, but the pandemic forced them to push the meeting to a fully online format in October 2020 and October 2021. This year, the annual meeting is planned to take place in October as a hybrid conference. He echoed Tajima’s statement that it became easier to invite more international speakers. Therefore, the annual meetings have started to highlight the opportunities for students to deliver presentations and meet international researchers around the world. He pointed out that the Physics Society of the Philippines is quite small compared to neighboring societies but still maintains vibrant activities. As everything is held in English, he considers that it would be quite easy for the regional workshops and conferences to be integrated into APPCs.

Nguyen Quang Liem explained that the Vietnam Physical Society organizes two international conferences: one is the biennial international workshop for material science and nanotechnology, and the other is the international conference on optics, spectroscopy, and applications. Each conference is attended by 500 participants from 30 countries including Japan, Korea, and European countries. Once in 2016, council members Gui-Lu Long and Fu-Jen Kao visited Vietnam. Another large-scale conference is organized by the Vietnam Physical Society every 5 years, while division-like smaller societies for solid-state physics, optics, education, and material science, under the Vietnam Physical Society, actively organize their conferences every year by inviting international speakers. The Vietnam Physical Society organized APPC9 in the past. In the forthcoming years after the pandemic is quelled, they might invite more participants from AAPPS to their conferences. Liem hopes such joint conferences will stimulate more interesting ideas to come and more collaborations to develop. The new council member Vu Dinh Lam, who represents the Vietnam Physical Society, will work more actively on this matter.

(7-1) Je-Geun Park, the chair of the Division of Condensed Matter Physics (DCMP) reported on the activities of the division. The division was established in early 2021 as the newest among four AAPPS divisions. Park and two vice chairs lead the seven other ordinary members of the executive committee. Several announcements are posted on the website regularly and they make their own flyers to publicize the activities. DCMP is one of the largest divisions under AAPPS and worked for successful programs at APPC15. The division will hold a separate meeting (AC2MP2022) in November 2022, under the support of the Institute of Material Science of Tohoku University. This time a hybrid format is much more emphasized than in-person. The division publishes newsletters electronically twice a year. The election of the chair and vice chair of the second DCMP team (term 2023–2024) is planned for September 2022. The division has been expanding through the joining of new members, such as AIP. Park requested the presidents of member societies to contact him or any exco members if any member society is interested in joining the DCMP.

(7-2) Weiping Liu, the chair of the Division of Nuclear Physics (DNP), gave a quick introduction to recent progress of the division. The Asia Nuclear Physics Association (ANPhA) was established in 2009 and the meetings have been held every year. This year’s meeting was held still online. Nevertheless, they have been making real progress through good interactions with not only division members but also counterpart researchers from Europe and the USA. A review article on the past 10 years of ANPhA was published in 2020. The new chair of the division will be elected very soon.

Nuclear physics is featured by large-scale facilities, traditionally concentrated in Japan, Korea, China, Australia, and India. Many large facilities mainly conduct basic research while small facilities conduct application-oriented research, and some others are under construction. A couple of symposiums are held every year, associated with the board meeting of ANPhA. This year’s meeting was held in conjunction with APPC15 and followed by introductions of representatives from different continents. Those kinds of activities were significantly impacted by the pandemic, and most of the activities were conducted in a hybrid fashion. The foresight program of the National Science Foundation’s A3 grant has been launched, and researchers from China, Korea, and Japan exchange very often. The activities supported by ANPhA include online colloquiums, nominations for the CN Yang Award, and a number of events for young scientists. Liu will deliver a plenary talk at APPC15.

Many large-scale facilities in Japan are in operation, while a huge project named RAON is in progress in Korea that is aiming at having the first beam in 2 years. The large facility CiADS in China near Hong Kong is nearly finishing its construction. The highlight of Asian facilities is Underground Nuclear Astrophysics in China (JUNA) for nuclear astrophysics. The facility is located 2400-meter-deep underground, and experiments to simulate nuclear reactions inside stars have been conducted. The progress is very smooth since the startup meeting held in 2013. To summarize, activities of Asia-Pacific nuclear physics are vibrant along with a huge number of collaborations with people from Asia Pacific and Europe.

Yokoyama asked whether any theorists are involved in the division. Liu stated that not only experimentalists but also theorists are involved. The construction plan heavily depends on theoretical calculations of reactions, which also guide experimental plans.

(7-3) Sang Pyo Kim, the chair of the Division of Astrophysics, Cosmology and Gravitation (DACG), reported on the recent activities of the division. The third term of the DACG exco started in 2021 and continues to 2023. Many activities have been organized and endorsed by the division. The annual workshop organized by the division was held in October 2021 in a hybrid format, which was attended by on-site participants from Korea and online participants from abroad. The LeCosPA symposium was organized by the Physical Society located in Taipei, and the International Conference on General Relativity and Gravitation (GR) was hosted by the Chinese Physical Society, Beijing. The latter was held in July 2022 as a hybrid event and was attended by more than 1100 participants from 52 countries, including 300 on-site participants from China. At APPC15, two plenary speakers recommended by the division and the winner of the CN Yang Award will deliver talks. The DACG Annual Meeting is planned to take place in November 2022. The 16th Dark Side of the Universe Workshop is scheduled for December 2022, which will be the first on-site conference since the pandemic.

Restoration of on-site activities and the rebuilding of the organization are areas of focus for DACG. S.P. Kim stated that he is undertaking an extension of the DACG exco and has already invited exco members from Singapore and Vietnam.

(7-4) Baonian Wan, the chair of the Division of Plasma Physics (DPP), reported on the most important activities of this year. The division will submit the activity report for the last 2 years to *AB*. This year, the division organized the plasma sessions at APPC15, where the division contributed with three plenary speakers and 100 presentations in three sub-sessions. The division nominated a candidate for the CN Yang Award, who was unfortunately not a winner, and the division chair was involved in the selection procedures. The 6th DPP Annual Meeting will be held in October 2022 as an online conference, for which the program is ready and registration has opened. At the DDP meeting, several prizes will be given, such as the Chandrasekhar Prize, Plasma Innovation Prize, the Young Researcher Award, and the Young Scientist and Student Awards. Wan’s term as the division chair will end in October and the next chair was elected from India.

The division publishes *Reviews of Modern Plasma Physics*. They have made great efforts to increase the number of publications and to improve the low impact factors. Already 20 review papers were published this year, which means that there was great progress as compared to prior years. The division collaborates with the Plasma Divisions of the APS and EPS and sends representatives to the joint program committee of their conferences.

Yokoyama commented that DPP has a legal entity registered in Japan, which is different from the other divisions. Wan responded that DPP is operated by the CEO with the board of directors.

Gui-Lu Long sent a message to the division chairs via chat: “You are invited to submit the summary of your presentation to *AB*. We will publish them in the News and Views section”. Yokoyama added that he met the president-elect of IUPAP, who showed interest in sharing AAPPS local news with them. This is an example of the importance of enriching the News and Views section to publish more society news in greater detail. Long sent another message to all participants: ”Please encourage your members to submit high-quality original research articles and review articles to *AB*. They will be reviewed and published after passing through the reviewing process. The estimated impact factor of *AB* should be more than 7.”

(8) Yokoyama stated that the Physical Society of Hong Kong has expressed their willingness to host a council meeting in person. However, he is afraid that the next meeting will have to be held online again by the end of this year. The agenda will include joining of the Physics Society of Iran and discussions regarding future APPCs.

Lastly, Yokoyama expressed his thanks to the AAPPS secretary and staff members from APCTP for their arrangement of all four meetings held today and closed the meeting.

## Report on the 11th AAPPS Ordinary General Meeting by AAPPS

The 11th Ordinary General Meeting (OGM) of the Association of Asia Pacific Physical Societies (AAPPS) was held in a hybrid format from 1:04 p.m. to 3:27 p.m. (UTC+9hr) on August 21, 2022. The in-person venue was the physics department’s faculty meeting room at Yonsei University, Seoul, which was connected online via a Zoom session hosted by the Asia Pacific Center for Theoretical Physics (APCTP). The participants were Jun'ichi Yokoyama (president, in person), Hyoung Joon Choi (vice president, in person), Nobuko Naka (secretary), Keun-Young Kim (treasurer, in person), and official representatives of member societies Kirrily Rule (proxy of Sven Rogge, Australian Institute of Physics (AIP)), Gui-Lu Long (ex officio member as a former president, editor-in-chief, and proxy of Jie Zhang, the Chinese Physical Society, Beijing), Xun-Li Wang (president of the Physical Society of Hong Kong), Vandana Nanal (proxy of S. Ramakrishnan, Indian Physics Association), Setsuko Tajima (president of the Physical Society of Japan (JPS)), Toshiro Hiramoto (president of the Japan Society of Applied Physics (JSAP)), Akira Yamada (proxy of Toshiro Hiramoto for the election, JSAP), Tae Won Noh (president of the Korean Physical Society (KPS), in person), Tou Teck Yong (president of the Malaysian Institute of Physics), Michael Francis Ian Vega II (proxy of Elmer Estacio, Physics Society of the Philippines), Rajdeep Singh Rawat (proxy of Christian Kurtsiefer, Institute of Physics Singapore), Ying-Jer Kao (president of the Physical Society located in Taipei), Bobomurat Ahmedov (proxy of Kadyr Gulamov, Council of Uzbekistan Physicists), and Nguyen Quang Liem (proxy of Nguyen Dai Hung, Vietnam Physical Society). Present as observers were council members Mio Murao (JPS), Kurunathan Ratnavelu (Malaysian Institute of Physics), Kadyr Gulamov (president of the Council of Uzbekistan Physicists, in person), Yunkyu Bang (president of APCTP, in person), Jae-Hyung Jeon (executive director of APCTP, in person), Mirim Lee (academic support staff, APCTP, in person), Sojin Park (academic support staff, APCTP, in person), and Kyeongtak Ryu (academic support staff, APCTP, in person). Official representatives T. A. Kozhamkulov (president of the Kazakh Physical Society), Nilam Shrestha Pradhan (president of the Nepal Physical Society), Riaz Ahmed (president of the Pakistan Physical Society), and Boonrucksar Soonthornthum (president of the Thai Physics Society) were absent.

(1) Secretary Naka reported the presence of 13 official representatives (including proxies) out of 20 member societies. All these official representatives present were eligible to vote and the simple majority was confirmed as seven. The minutes of the 10th Ordinary General Meeting were approved.

(2) Yokoyama opened the 11th Ordinary General Meeting (OGM) and welcomed the participants. All participants introduced themselves.

(3) The agenda was adopted as prepared by the president.

(4) Naka explained the procedure for the election of new council members. At the 47th Council Meeting held on December 18, 2021, the size of the next council was determined as 15(+1+1+ex officio). A call for nominations was made to the representatives of member societies and 18 nominations were received from 13 member societies. The list of the candidates and their brief CVs were circulated for the secondment. The final slate was made, which comprised of all 18 candidates seconded at least by one other member society. Naka explained that a new position, “associate council member,” was proposed and approved at the same council meeting. For more member societies to join discussions and express their opinions at council meetings, the council decided to invite one representative as an associate council member from each society that does not send council members. Associate council members have no voting rights.

The slate of 18 candidates for the next council was confirmed. Naka reported that she sent a notice on Clause 7.3 of the Constitution that member societies with unpaid fees have no voting rights at the OGM. It was confirmed that all 13 official representatives present have voting rights. Subsequently, Naka explained the election rules and conveyed a question about a tie vote. Yokoyama suggested the acceptance of both two candidates in light of the possible extension of the council size in near future, which was the next item on the agenda of this meeting. Yunkyu Bang commented that, although he is an observer and not an authority at this meeting, there is some international tradition to take the older candidate. Yokoyama stated that he would rather choose a younger candidate in such an occasion. Xun-Li Wang stated that it seems to be a more hypothetical situation and we should just proceed. He added that this is a good point for the council to consider and the rule to break a tie should be formulated. It was agreed to conduct the first round and to see the result.

Naka requested the official representatives to cast votes for up to 15 candidates in the first round. All 13 voting ballots collected were confirmed to be valid with the number of selected candidates not exceeding 15. According to the rank based on the number of votes received by each candidate, the top 15 candidates were declared elected after confirming that they received more than the simple majority. A second, tiebreaking round was not needed because the candidate in the 16th place received fewer votes than the candidate in the 15th place. The names and societies of new council members elected are listed below in alphabetical order of the name of member societies.

<The list of new council members elected (term 2023-2025)>

1. Nicole F. BELL (Australian Institute of Physics)

2. Xiudong SUN (the Chinese Physical Society, Beijing)

3. Tao XIANG (the Chinese Physical Society, Beijing)

4. Ruiqin ZHANG (the Physical Society of Hong Kong)

5. Mio MURAO (the Physical Society of Japan)

6. SHEN Qing (the Japan Society of Applied Physics)

7. Hyoung Joon CHOI (the Korean Physical Society)

8. Jae-Hyung JEON (the Korean Physical Society)

9. Keun-Young KIM (the Korean Physical Society)

10. Kurunathan RATNAVELU (Malaysian Institute of Physics)

11. Narayan Prasad CHAPAGAIN (Nepal Physical Society)

12. Rajdeep Singh RAWAT (Institute of Physics Singapore)

13. Meng-Fan LUO (the Physical Society located in Taipei)

14. VU Dinh Lam (Vietnam Physical Society)

15. Kadyr GULAMOV (Council of Uzbekistan Physicists)

Naka expressed her gratitude to the official representatives of the member societies for their active participation in the nomination and election processes, and to Ms. Dayoung Yang and her team for the excellent arrangement of election forms and email correspondences.

(5) Yokoyama proposed a possible amendment to the Constitution of AAPPS.

At the 49th Council Meeting held on July 28, 2022, the council discussed that it would be appropriate to expand the size of the council as the number of our member societies has been increasing and this trend is expected to continue. Subsequently, Yokoyama received a written proposal from Prof. Ying-Jer Kao, president of the Physical Society located in Taipei, who attended the council meeting as the proxy to Prof. Meng-Fan Luo, to increase the number of council members to broaden the representation. Yokoyama explained that its upper limit is currently determined in Clause 5.3 as 15 for those elected at the OGM, and the change needs an amendment to the Constitution. Ying-Jer Kao added that he was the only one that could make the proposal among those who were present at the meeting.

As described in Clause 9 of the Constitution, there are three steps to amend the Constitution. Yokoyama explained that a council member is not an authority to propose an amendment. The first step, defined by Clause 9.1, i.e., proposal by a member, has been filled by the message from Prof. Kao. After approval by the council, the president informed the representatives of member societies of this proposal on August 4, following Clause 9.2. Clause 9.3 states that a change in the Constitution shall require a two-thirds majority vote at a General Meeting.

Yokoyama explained that the motion is to change the phrase, ”no more than 15” to “no more than 20” in Clause 5.3, as unanimously agreed by the council. He added that the amendment, even if accepted, does not mean an immediate increase in the number of council members to 20 for the term starting from 2026. The number of new council members would be determined, within the lower and upper limits, by the outgoing council.

Yokoyama proposed that we should exchange opinions and then proceed with a vote. (See Appendix 1 for details of the discussion.) After a discussion, an open vote was proposed and conducted. The result was 7 for the change, 3 against the change, and 1 staying vote. Because a two-thirds majority was not obtained, the motion for amendment to the Constitution was rejected.

Yokoyama finally commented that there are several things to be considered about the Constitution. There are simple typos, which we would like to correct. Some timelines are too long because airmail was the most rapid means of communication at the time of the Constitution’s formulation. The mutual understanding for the council members to serve up to three terms should be explicitly stated in the Constitution. Yokoyama requested that the presidents of member societies read the Constitution and propose amendments if necessary. He noted once again that only the members of the OGM (i.e., representatives of the member societies of AAPPS) can propose an amendment to the Constitution.

(6) Yokoyama reported on the activities of the past 3 years (see Appendix 2)

Yokoyama summarized his report that we are growing in terms of the numbers of member societies, divisions, and meetings in AAPPS, although most of the activities during this term (2020–2022) were conducted on online platforms. There are some remaining issues, which were not realized in this term. The most important one is the establishment of a legal entity of AAPPS, for which Vice President Choi has been investigating the possibility in Korea. Yokoyama stated that the financial vulnerability of AAPPS has been largely helped by the APCTP, both via direct and in-kind supports. Cooperation with the European Physical Society (EPS) for the next Asia Europe Physics Summit (ASEPS) is also a remaining issue. Yokoyama emphasized that these cooperative societies truly help energize the activities of AAPPS. He concluded the report by stating that our goal is to work together to make our world more prosperous and peaceful through communications, research, and education in physics.

Xun-Li Wang appreciated the leadership of President Yokoyama and the council during this term. In the last few years of the pandemic, the Physical Society of Hong Kong was more connected with other physical societies, through AAPPS. He hopes AAPPS continues to help the community grow and increases its activities. Tou Teck Yong also expressed his gratitude to Yokoyama for his great efforts.

(7) Treasurer Keun-Young Kim reported on the financial status of AAPPS. (Refer to item 6 of the 50th Council Meeting.)

(8) Gui-Lu Long, the editor-in-chief of the *AAPPS Bulletin* (*AB*), reported on the current status of *AB*. (Refer to item 7 of the 50th Council Meeting.)

(9) Jae-Hyoung Jeon gave a report from APCTP as the executive director. He explained that AAPPS was one of the global associations that supported the foundation of APCTP in 1996. In 2013, the center moved from Seoul to Pohang and has been hosting the headquarters of AAPPS since 2016.

The center currently has 17 member entities and 33 partnership institutions. Its mission is to act as a hub center for academic activities. The center supports as many as 70 conferences per year and provides in-house research programs and education and training for young scientists, including Junior Research Groups, Young Scientist Training Programs, and Senior Advisory Groups. For science diplomacy, the center cooperates with AAPPS, the Asia Pacific Economic Cooperation (APEC), the International Union of Pure and Applied Physics (IUPAP), the American Association for the Advancement of Science (AAAS), and *AB* for publication.

Since 2004, APCTP has provided support for meetings for AAPPS ($72,000 USD), has supported APPCs ($112,000 USD), and has supported *AB* ($231,970 USD). The support for division activities started in 2017 and amounts to $23,000 USD per year. In addition, APCTP has been providing the monetary prize of $1000 USD for each winner of the AAPPS-APCTP CN Yang Award and provided support to renovate the *AB* website.

Kadyr Gulamov wondered how the center manages to organize 70 conferences every year. Yunku Bang clarified that the number includes conferences and colloquiums, not only organized but also endorsed by APCTP.

(10) Yokoyama thanked the official representatives of member societies for their participation and closed the meeting.

## Appendix 1

### Discussion on the council size

- Bobomurat Ahmedov wondered why we have an even number. Yokoyama answered that 20 is the possible largest number and there are other members with a voting right in the council, such as the ex officio.
- Michael Francis Ian Vega II was trying to understand the background of the proposal and the broader rationale of the change. He was curious about whether there was a sentiment that the current number of council members represents a severe underrepresentation among member societies. He stated that the increase in the number of AAPPS member societies does not necessarily imply underrepresentation. Yokoyama responded that the reason for the proposal is to appropriately represent member societies whose number has been increasing. Currently, some member societies are sending more than one council member.
- Tou Teck Yong expressed his concern that it would become financially more difficult for a small society to host a council meeting and invite all of the council members, as the Malaysian Institute of Physics did on the occasion of APPC14. He imagines that a small society with a limited budget would never be able to host council meetings unless the council members pay themselves to travel to the venue. Although he appreciates that APPCs are successfully organized mostly by financially stronger, active, and supportive member societies, he is not sure whether the AAPPS council assures to help smaller societies. Tou pointed out that we might have a situation where only four countries or regions occupy the whole council.
- Tou stated that he does not think it practical to have more council members than currently. Tou wondered why five more council members are needed for only one meeting, where a vote occurs only every 3 years. Yokoyama clarified that this situation describes an OGM, where official representatives gather once every 3 years, whereas the council itself has many more meetings.
- Vandana Nanal focused on two points. First, due to travel restrictions and other difficulties for council members, hybrid meetings ensure wider representation. Second, regarding the size of the council, the representation of member societies in the AAPPS council appears to be not very balanced. The reason why member societies want to be a part of the council is that they want to be more interactive regarding the policies discussed in the meetings with major societies in the Asia Pacific region. She was unsure if this could be achieved simply by increasing the number of council members. As mentioned earlier (See agenda item 4), at least through the scheme of associate membership, we will be able to keep the whole council balanced without expanding its size. She suggested having associate members from all member societies. Yokoyama agreed that better representation could be realized by continuing hybrid or online meetings with the inclusion of associate council members.
- Kirrily Rule pointed out that increasing the number of council members to 20 would not automatically give better representation to the smaller societies. She stated that the whole point of increasing the number is to give fair representation; consequently, the constitution would need to be changed even further to state that at least one member from each member society should be represented at council meetings. Tou confirmed that presently, not all the member societies that pay their respective membership fee send a council member.
- Nanal commented that there was a discussion in the Indian Physics Association when they paid the membership fee. They want to be more associated with the Asia Pacific regional activities, and they are a member. However, the specific benefits of AAPPS membership are very difficult to explain. This will go back to the point that there is no fair representation in the council. Yokoyama welcomed the restoration of India to AAPPS with payment of all remaining membership fees. He explained that we did not receive any nomination from the Indian Physics Association although we sent the call and reminders. Yokoyama stated that active participation in the election is highly appreciated. Nanal responded that it would be now possible with an online format. She stated that there might have been some communication problems, but the community people will feel more involved if the Indian Physics Association would play a major role in AAPPS activities.
- Wang stated that he has trouble understanding the reason for increasing the number of council members. He always had the impression that the current scheme of the council is working quite well and he finds no rationale to increase the number of members. He added that we might fail to have 20 candidates, considering that only 18 candidates were nominated for 15 seats this time. Yokoyama responded that on the other hand, all those who are interested in joining the council will be accepted if we increase the number to 20. Wang agreed that that is also true.
- Rawat understands the point that the council should include representatives from as many societies as possible. He stated that we might probably need to qualify our tradition that both societies, which have paid or unpaid fees, can send a council member. He expressed his thoughts on the reason why a few major societies represent a high total percentage of the council. Those societies are actively participating and showing their presentations, by paying not only the membership fees but also through other contributions, as will be explained in the financial report. Namely, these five leading societies contribute strongly to the functioning of AAPPS, and their large representation in the council could be considered as real recognition of their respective societies’ contributions to AAPPS. As the Constitution was formulated 30 years ago, we need to think about its updates. One of the past drawbacks of the operation of AAPPS was that council meetings were held only on-site and participation from smaller member societies was not easy. A positive change is the possibility for online meetings, where we could be more inclusive. He summarizes that we will have to look into not only the number of council members but also several factors in the Constitution itself.
- Yokoyama stated that, as pointed out by Prof. Tou, a council consisting of members of only four societies is in principle possible but would never happen because an equal voting right is offered to each member society at the OGM. This is indeed observed in today’s election result (see item 4), where some member societies had sent more than one nomination and they were not fully elected. The current scheme is working well and the council concluded that just increasing the numbers should work.

## Appendix 2

### Presidential report

Yokoyama has been serving as the president of AAPPS since January 2020. The start of the term was just after the outbreak of Covid-19. Therefore, AAPPS activities were mostly restricted to online ones. So far, council meetings were held nine times (from the 43rd to the 50th Council Meetings, including the 44^th^: Parts I and II). Previously, the council meetings were held on-site and only once a year as it cost too much for council members to directly meet more frequently. Thanks to the development of the online platform, many more council meetings were held this term than previously.

At the beginning of Yokoyama’s term, there were 18 member societies. There are now 20 member societies, as Uzbekistan and Pakistan have now joined AAPPS. The Physics Society of Iran also expressed interest to join and will deliver a presentation at a future council meeting for approval of membership. In comparison, IUPAP has 60 member societies and AAPPS has a significant percentage of the physicists in the world. Yokoyama showed the list of officers, current council members, and their member societies and explained that five societies are providing extra support in addition to the membership fees.

The council organized the Asia Pacific Physical Societies’ Forum in November 2021, at which 14 member societies and the Council of Uzbekistan Physicists delivered presentations. India, Nepal, and the Philippines have also rejoined the activities of AAPPS.

The headquarters of AAPPS is hosted by and located at APCTP in Pohang, Korea. Yokoyama acknowledged support from APCTP to AAPPS and stated that this meeting is attended by Yunkyu Bang, the president of APCTP, and Jae-Hyung Jeon, the executive director of APCTP. The memorandum of understanding between APCTP and AAPPS was first signed in 2011 and has been automatically renewed every 5 years.

The main activities of AAPPS consist of publication of the *AAPPS Bulletin*, organization of APPCs and Asia Europe Physics Summit (ASEPS) meetings, selection and awarding of the CN Yang Award, and division activities.

The next APPC (APPC16) is planned to take place in Beijing in 2025. Yokoyama discussed with the European Physical Society (EPS) to resume a face-to-face meeting of ASEPS, which had been suspended due to the Covid-19 pandemic. The next ASEPS will be hosted in Europe.

In 2017, the previous council determined the rules on sponsorship, co-sponsorship, and endorsement of AAPPS activities, under the former President Long. Part of the rule will soon be promoted to the Code of Conduct, whose draft was approved at the 50th Council Meeting. AAPPS has provided support to the conferences held in Malaysia in 2021, Nepal in 2021, and Thailand in 2022. Some speakers were sent to these conferences from the AAPPS council and member societies. The main scope of the conference in Thailand was applied physics, and two plenary speakers were recommended by JSAP, which has been providing extra financial support to AAPPS activities. Yokoyama has so far attended 10 meetings as the president.

The CN Yang Awards are currently disbursed in partnership between AAPPS and APCTP every year. Yokoyama acknowledged APCTP for supporting the prize money for three awardees every year and holding ceremonies in the years between the APPCs. It has been pointed out that in order to attract a greater audience to the award ceremony, it may be better to have the ceremony at an annual meeting of the member society to which the awardee belongs. Yokoyama requested the official representatives to consider the feasibility of this change. He also stated that he welcomes opinions on how we should incorporate gender, geographical, and subject balances.

Sparked by Yokoyama’s participation at a meeting of the Physical Society located in Taipei, the AAPPS-XPS Award was proposed as a joint award for talented young researchers, where X represents the initial letter of the member society’s name. The joint award of the Physical Society located in Taipei launched as a pilot program and the first-year prizes were already provided to three researchers from the Physical Society located in Taipei. The winners received gold medal plates from AAPPS, which were donated by the AAPPS president, and monetary prizes from the Physical Society located in Taipei. At the 49th Council Meeting, the guidelines for establishing such an award were formulated and approved (the related material has been sent to the presidents of member societies by e-mail). JPS is also planning to establish a joint award in a different format. Yokoyama stated that these new joint awards will enhance the visibility of AAPPS, similarly to the IUPAP’s Early Career Scientist Prize (formally the Young Scientist Prize), which has spread the visibility of IUPAP among young researchers.

There were three divisions when Yokoyama’s term began. The Division of Plasma Physics (DPP) was the first division created under AAPPS and has its own review journal. The Division of Astrophysics, Cosmology and Gravitation (DACG) organizes annual conferences and schools. In the Division of Nuclear Physics (DNP), the Asian Nuclear Physics Association (ANPhA) acts as the division and conducts many activities. The Division of Condensed Matter Physics (DCMP) is the newest division, established in January 2021, which covers all the fields of condensed matter physics. AAPPS has a similar division structure as the 12 divisions of the EPS, but has not reached a stationary state yet. The foundation of the Division of Particles and Fields is underway and that of the Division of Computational Physics is under discussion.

Women-in-Physics activities are important in AAPPS. On Tuesday August 23, 2022, Women-in-Physics sessions will be held at APPC15. In some countries in Asia, such as Malaysia and Myanmar, the majority of physicists are women. Some of the members of DNP are organizing education programs to support physicists in Myanmar, who have difficulties under military rule.
